# The PPAR-γ antagonist GW9662 elicits differentiation of M2c-like cells and upregulation of the MerTK/Gas6 axis: a key role for PPAR-γ in human macrophage polarization

**DOI:** 10.1186/s12950-015-0081-4

**Published:** 2015-05-03

**Authors:** Gaetano Zizzo, Philip L Cohen

**Affiliations:** Temple Autoimmunity Center, Temple University, 3500 N. Broad Street, 19140 Philadelphia, PA USA; Department of Medicine, Section of Rheumatology, Temple University, 3322 N. Broad Street, 19140 Philadelphia, PA USA

**Keywords:** Macrophages, M2, Peroxisome Proliferator Activated Receptor-gamma (PPAR-γ), Liver X Receptor (LXR), Mer receptor Tyrosine Kinase (MerTK), Gas6, CD163

## Abstract

**Background:**

The nuclear receptors PPAR-γ and LXRs regulate macrophage lipid metabolism and macrophage mediated inflammation. We examined the influence of these molecules on macrophage alternative activation, with particular focus on differentiation of “M2c” anti-inflammatory cells.

**Methods:**

We cultured human monocytes in M0, M1, M2a or M2c macrophage differentiating conditions, in the presence or absence of PPAR-γ and LXR ligands. Flow cytometry was used to analyze membrane expression of phenotypic markers. Basal and LPS-stimulated production of soluble mediators was measured by ELISA. Efferocytosis assays were performed by coincubating monocytes/macrophages with apoptotic neutrophils.

**Results:**

We found that PPAR-γ inhibition, using the PPAR-γ antagonist GW9662, elicits differentiation of *M2c-like* (CD206^+^ CD163^+^ CD16^+^) cells and upregulation of the MerTK/Gas6 axis. Exposure of differentiating macrophages to IFN-γ, GM-CSF or LPS (M1 conditions), however, hampers GW9662 induction of MerTK and Gas6. When macrophages are differentiated with IL-4 (M2a conditions), addition of GW9662 results into an M2a (CD206^+^ CD209^+^ CD163^−^ MerTK^−^) to M2c (CD206^high^ CD209^−^ CD163^+^ MerTK^+^) polarization shift. Conversely, in the presence of dexamethasone (M2c conditions), the PPAR-γ agonist rosiglitazone attenuates CD163 and MerTK upregulation. The LXR agonist T0901317 induces MerTK independently of M2c polarization; indeed, CD206, CD163 and CD16 are downregulated. GW9662-differentiated *M2c-like* cells secrete high levels of Gas6 and low amounts of TNF-α and IL-10, mimicking dexamethasone effects *in vitro*. However, unlike conventional M2c cells, GW9662-differentiated cells do not show enhanced efferocytic ability.

**Conclusions:**

Our results provide new insights into the role of PPAR-γ and LXR receptors in human macrophage activation and reveal the existence of different patterns regulating MerTK expression. Unexpectedly, PPAR-γ appears to negatively control the expansion of a discrete subset of *M2c-like* anti-inflammatory macrophages.

## Background

Macrophages are heterogeneous cells endowed with great plasticity. Traditionally, macrophage activation broadly divides these cells into: “classical” or “M1” oriented, thought to mediate innate immune responses against pathogens and recruitment of adaptive responses through antigen processing and presentation; and “alternative” or “M2” oriented, important in resolution of inflammation, tissue repair and homeostasis [1,2]. M1 macrophages are induced by interferon-gamma (IFN-γ), lipopolysaccharide (LPS), tumor necrosis factor-alpha (TNF-α) and/or granulocyte macrophage colony stimulating factor (GM-CSF), and are responsible for pathogen phagocytosis, oxidative burst and intracellular killing, as well as for stimulation of T helper (Th) 1 and Th17 responses [1-3]. M2 macrophages are further subdivided into several subsets: M2a [CD206/mannose receptor^+^ CD209/DC-SIGN^+^ CD163^−^ CD16^−^ Mer receptor tyrosine kinase (MerTK)^−^], induced by interleukin (IL)-4 and/or IL-13, prone to endocytosis, wound-healing, and stimulation of Th2 response; M2b, induced by immune complexes, characterized by a mixed production of IL-10 and inflammatory cytokines (primarily described in mice); and M2c (CD206^high^ CD209^−^ CD163^+^ CD16^+^ MerTK^+^), induced by macrophage colony stimulating factor (M-CSF) plus IL-10 or by glucocorticoids, involved in anti-inflammatory responses and designated to the clearance of apoptotic cells (ACs) through MerTK and its ligands Growth arrest-specific 6 (Gas6) and Protein S [1-5].

The activation state of macrophages is closely related to their metabolic state. While in acutely inflamed hypoxic tissues, anaerobic glycolysis fuels the microbicidal program of M1 macrophages [6], aerobic metabolism might instead fuel M2 activation, thereby allowing long-term macrophage responses best suited for helminth infections or other chronic conditions [7,8]. Lipid handling and metabolism are associated with immune regulatory macrophage responses, finely controlled by the nuclear receptor superfamily members peroxisome proliferator activated receptor-gamma (PPAR-γ) and liver X receptors (LXRs). These transcription factors are tightly interlinked, and act as heterodimers with the same partner, the retinoid X receptor RXR-α [9]. PPAR-γ can be activated by purely metabolic signals [i.e., polyunsaturated fatty acids and lipoproteins, such as hydroxyoctadecadienoic acids (HODEs), hydroxyeicosatetraenoic acids (HETEs) and oxidized low density lipoproteins (oxLDLs)] [10,11], by molecules at the crossroads between lipid metabolism and inflammation [i.e., eicosanoids, such as 15-deoxy-delta-12,14-prostaglandin J_2_] [11], or by purely immunologic signals (i.e., cytokines, such as IL-4, IL-13, and GM-CSF] [12-17]. In turn, PPAR-γ activation results in lipid uptake through the scavenger receptor CD36 and β-oxidation of fatty acids [7,9], modulation of the phospholipase A2/cyclooxygenase-2 axis [18], and macrophage differentiation *via* STAT-6 into M2a cells [7,8,12,17,19]. LXRs are cholesterol sensors, induced by several oxysterols and by ACs [9,20]. LXR activation results in a positive feedback loop driving further uptake of ACs through the induction of MerTK [20], inhibition of lipoprotein uptake [21] and reverse cholesterol transport from macrophages to high density lipoproteins [9].

PPAR-γ and LXR activities are finely coordinated. PPAR-γ is in fact able to activate LXRs [9]. The integration between these two networks ensures a link between lipid uptake and cholesterol efflux, thereby protecting macrophages from lipid overload and conversion to foam cells. Coordination between PPAR-γ and LXRs is also explained by similar functions in regard to scavenging of modified lipoproteins, ACs and pathogens, and by the fact that both receptors are involved in modulatory responses, including SUMOylation-dependent transrepression of NF-κB [22] and inhibition of several inflammatory genes [9,23-25]. On the other hand, in certain conditions, PPAR-γ and LXRs exert opposing roles. In M2a macrophages, IL-4 stimulates the expression of PPAR-γ as well as the production of its ligands 13-HODE and 15-HETE through the induction of 12/15-lipoxygenase (15-LOX) [12]; however, 15-LOX activation also results in LXR-α downregulation, so that in this M2 subtype, PPAR-γ is strongly induced but LXR-α is inhibited [26]. Ultimately, PPAR-γ and LXRs appear to regulate analogous cell functions by controlling different molecular pathways. The PPAR-γ network includes a spectrum of scavenger receptors (i.e., class B receptors SR-BI and CD36) [12,13,19], apoptotic receptors (i.e., CD36, thrombospondin-1, and transglutaminase-2, all involved in β_3_ integrin mediated pathways) [26-29] and pathogen receptors (i.e., CD36, dectin-1) [19,30] which is different from the panel of receptors upregulated by LXRs (i.e., class A receptor MARCO, MerTK, and apoptosis inhibitory factor AIM/SP-α/Api6, respectively) [20,31].

In the present study, we investigated the effects of PPAR-γ and LXRs in differentiation of M2c macrophages and induction of the MerTK/Gas6 axis. We found that PPAR-γ obstructs whereas LXRs promote MerTK upregulation. Importantly, MerTK expression induced by the PPAR-γ antagonist GW9662 is associated with M2c polarization, whereas LXR induction of MerTK occurs regardless of M2c phenotype acquisition. GW9662-driven *M2c-like* cells also release high amounts of Gas6 and low levels of TNF-α, but differ from conventional M2c cells by not showing enhanced clearance of ACs. These data contribute to better define the role of PPAR-γ and LXRs in human macrophage activation, and point out the existence of distinct regulation patterns for MerTK expression. The unexpected finding that PPAR-γ negatively controls the expansion of a discrete subset of anti-inflammatory macrophages may also have clinical implications.

## Methods

### Cell cultures

Monocytes from buffy coats of healthy blood donors were isolated by Ficoll-Paque™ Plus gradient (GE Healthcare Life Sciences, Pittsburgh, PA, USA) and magnetic separation, using a kit for human monocyte enrichment by negative selection (EasySep™, StemCell Technologies, Vancouver, BC, Canada), according to the manufacturer’s instruction. CD14+ cells were cultured at 0.8×10^6^ cells/ml in non-tissue culture treated 24-well plates in X-Vivo™15 medium (Lonza, Walkersville, MD, USA) at 37°C in 5% CO_2_ for 4 days, in the presence of rosiglitazone (PPAR-γ agonist, 0.1-10 μM), GW9662 (PPAR-γ antagonist, 0.01-10 μM) or T0901317 (LXR agonist, 0.001-1 μM) (Cayman Chemical, Ann Arbor, MI, USA). GW9662 (lot 0417082–20) was reconstituted in ethanol 2 mg/ml. T0901317 and rosiglitazone were reconstituted in dimethylsulfoxide (DMSO) 5 and 10 mg/ml, respectively. Serial dilutions were performed using culture medium. Cells were ultimately exposed to working solutions containing non-cytotoxic amounts of ethanol or DMSO (≤0.1%). In some experiments using high concentrations of reagent (i.e., rosiglitazone 50–100 μM) and vehicle (i.e., DMSO > 0.15%), vehicle controls were included. When specified, cells were differentiated in the presence of GM-CSF 100 ng/ml (Peprotech, Rocky Hill, NJ, USA) or IFN-γ 2.5 ng/ml (R&D Systems, Minneapolis, MN, USA) for M1 differentiation, IL-4 20 ng/ml (Novus Biologicals, Littleton, CO, USA) for M2a differentiation, and dexamethasone 100 nM (Sigma-Aldrich, St. Louis, MO, USA) for M2c differentiation. In some experiments, cells were coincubated with low doses of LPS (50 ng/ml; extracted from E. Coli 026:B6, Sigma-Aldrich) to stimulate cytokine secretion. After differentiation, cells were incubated for 20 minutes at 37°C with a detaching buffer containing EDTA 10 mM and lidocaine 15 mM in sterile Phosphate Buffered Saline (PBS). Cells were then washed and harvested by centrifugation. Pellets were resuspended in PBS containing 2% bovine serum albumin and freshly analyzed by flow cytometry. Supernatants were collected and immediately stored at −20°C before being tested by ELISA. Participants gave informed consent to donate their blood samples. The study was approved by the Institutional Review Board of Temple University.

### Flow cytometry

Phenotypic analysis was carried out on cultured monocytes/macrophages by using the following mouse monoclonal antibodies: anti-CD14 (PE-Cy7), anti-CD163 (APC), anti-CD206 (APC-Cy7), anti-CD209 (PerCP-Cy5.5) (Biolegend, San Diego, CA, USA); anti-CD16 (APC-Cy7) (BD Biosciences, San Jose, CA, USA); and anti-MerTK (clone 125518; PE) (R&D Systems). MerTK expression was evaluated using appropriate PE-labeled isotype control (Biolegend). Cells were analyzed using FACSCalibur™ (BD Biosciences) and FlowJo software (Tree Star, Ashland, OR, USA).

### ELISA

Gas6, IL-10 and TNF-α levels were measured in supernatants of cell cultures using sandwich ELISA according to standard procedure [32]. Briefly, 96-well plates were precoated overnight with a capture antibody. Samples from cell culture supernatants were applied to precoated plates in duplicate. Serial dilutions of purified recombinant rhGas6 (R&D Systems) were used to construct a standard curve. Blank wells received serum-free X-Vivo™15 medium. A purified goat polyclonal anti-human Gas6 antibody (R&D Systems) was used for capture. Biotinylated goat polyclonal anti-human Gas6 antibody (R&D Systems), followed by HRP-conjugated streptavidin (Biolegend), was used for detection. The plates were developed with 3,3′,5,5′-tetramethylbenzidine substrate. The reaction was stopped with 2 N sulfuric acid. Absorbance was detected at 450 nm and read with a reference wavelength set at 570 nm using a VersaMAX ELISA microplate reader (Molecular Devices, Sunnyvale, CA, USA). The optical density for each point was the average of duplicate samples. Concentrations were determined using SoftMax software (Molecular Devices) by applying four-parameter logistic regression to the standard curve. IL-10 and TNF-α levels were measured using human IL-10 ELISA MAX Standard kit and TNF-α ELISA MAX Standard kit (Biolegend), following the manufacturer’s instructions.

### Apoptotic cell phagocytosis assay

Human neutrophils were isolated from Ficoll-Hypaque pellets through dextran erythrocyte sedimentation and lysis of contaminating erythrocytes by incubation with ice-cold ammonium chloride (0.15 M) and potassium bicarbonate (0.01 M) solution. Neutrophils were resuspended at 1×10^6^ cell/ml in 10% fetal bovine serum (FBS) - RPMI 1640 medium, labeled with 2.5 μM carboxyfluorescein succinimidyl ester (CFSE; Sigma-Aldrich), and incubated for 20 hours at 37°C in 5% CO_2_. Allophycocyanin-conjugated annexin V (BD Biosciences) and propidium iodide (PI; Sigma-Aldrich) were used to measure apoptosis by flow cytometry. The composition of neutrophils routinely obtained after incubation was 77.50 ± 10.05% for early apoptotic cells (ACs) (annexin V^+^ PI^+^), 4.59 ± 2.34% for late ACs (annexin V^+^ PI^+^), and 0.38 ± 0.29% for necrotic cells (annexin V^+^ PI^+^). Macrophages were differentiated in X-Vivo™15 medium supplemented with 10% human AB serum, 5% FBS, L-glutamine, penicillin and streptomycin, in the presence or absence of GW9662 (10 μM), for 6 days. In some experiments, cells were cultured in the presence of IL-4 (20 ng/ml) to obtain M2a macrophages. On day 6, apoptotic neutrophils were added for 30 minutes to cultured macrophages at a 5:1 ratio. Flow cytometry was used to quantify percentages of CD14-labeled macrophages that phagocytosed CFSE-labeled ACs.

### Statistical analysis

Data are expressed as mean ± SEM. Statistical significance among different cell treatments was assessed by Student’s paired *t*-test, or one-way repeated measures ANOVA with Newman-Keuls multiple comparisons test if more than two treatment groups were compared. Statistical significance was defined as P <0.05. Analysis and graphing were performed using Prism™ software (GraphPad Software, La Jolla, CA, USA).

## Results

### The PPAR-γ antagonist GW9662 inhibits IL-4-driven macrophage alternative activation by inducing a phenotypic M2a-to-M2c switch

**Figure 1:**
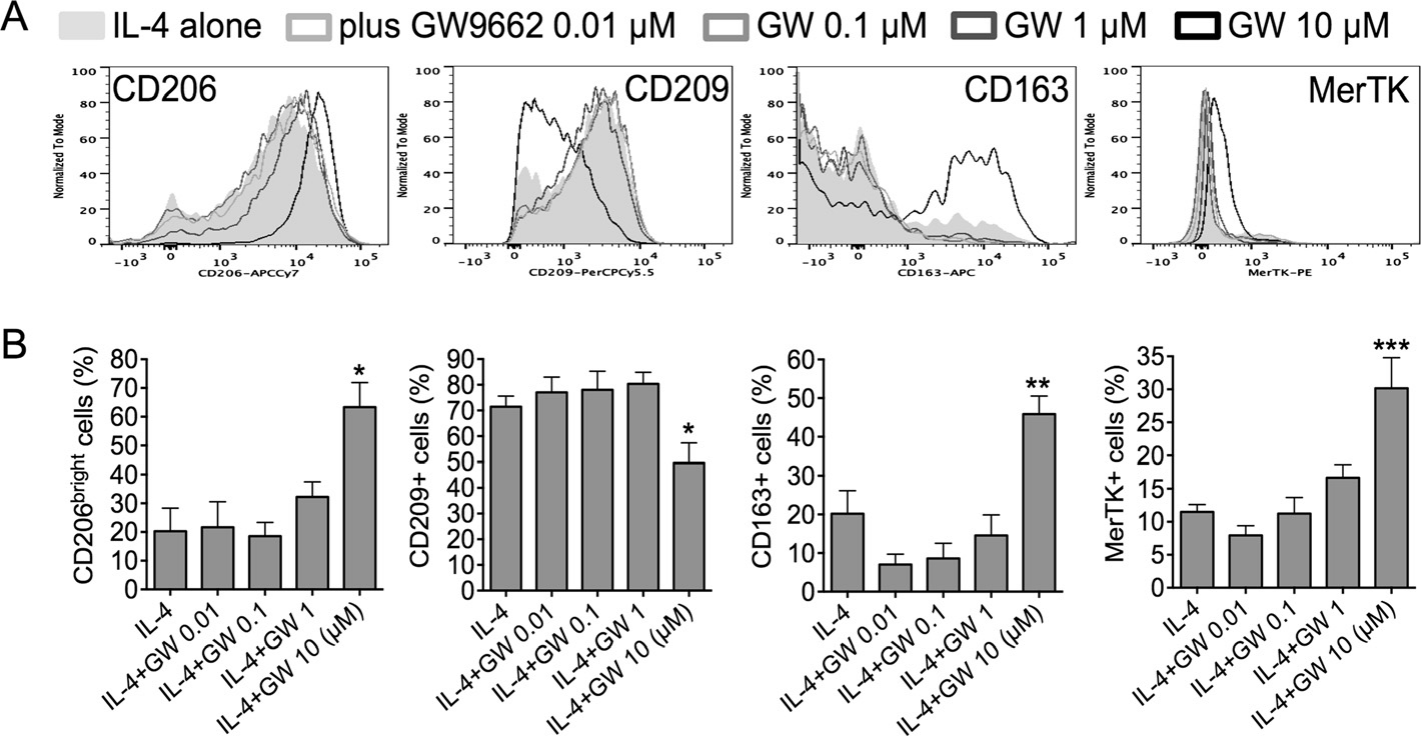
GW9662 inhibits IL-4-driven alternative activation by inducing a phenotypic M2a-to-M2c switch. **(A-B)** Human monocytes were sorted from healthy PBMCs through negative selection magnetic beads and cultured in serum-free medium in the presence of IL-4 (20 ng/ml; M2a differentiation), with or without the PPAR-γ antagonist GW9662 (0.01-10 μM), for 4 days. Expression of the M2 markers CD206 (mannose receptor), CD209 (DC-SIGN), CD163 and MerTK was measured by flow cytometry. **(B)** Pooled data are represented as mean values ± SEM. Analysis was performed using one-way repeated measures ANOVA with Newman-Keuls multiple comparisons test. *P < 0.05; **P < 0.01; ***P < 0.001. Data are representative of three independent experiments.

Many of IL-4’s effects on macrophage alternative (M2) differentiation are mediated by PPAR-γ via STAT6 [7,8,12,17,19]. IL-4 specifically induces the “M2a” phenotype, which is characterized by expression of the universal M2 marker CD206, high levels of CD209 and low membrane expression of CD163 and MerTK [4]. IL-4 hinders induction of the “M2c” phenotype, which is instead characterized by high levels of CD206, CD163 and MerTK and low levels of CD209 [4,33]. Here, we used flow cytometry to assess the effects of PPAR-γ inhibition on the phenotype of IL-4 exposed cells. Differentiation of monocytes/macrophages in the presence of IL-4 and the PPAR-γ antagonist GW9662 resulted in brighter expression of CD206, inhibition of CD209 induction, and upregulation of CD163 and MerTK. GW9662 effects were dose-dependent (Figures 1A-B). Thus, blocking PPAR-γ activation during M2a differentiation provokes a phenotypic switch from M2a (CD206^+^ CD209^+^ CD163^−^ MerTK^−^) to M2c (CD206^high^ CD209^−^ CD163^+^ MerTK^+^) cells.

### M1 polarizing agents IFN-γ and GM-CSF act to oppose GW9662 effects on macrophage phenotype

**Figure 2:**
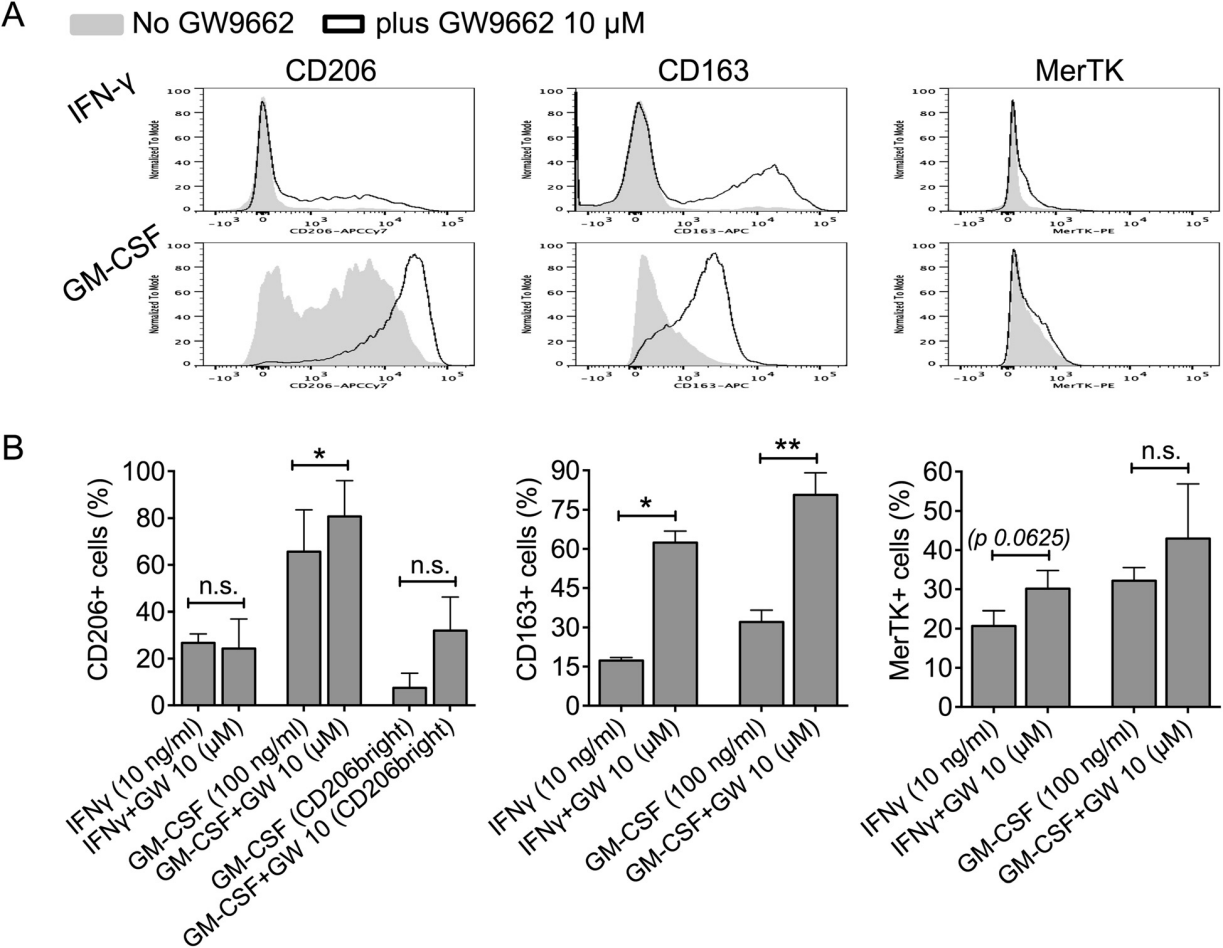
M1 stimulation with IFN-γ or GM-CSF opposes GW9662 effects on macrophage phenotype. **(A-B)** Healthy monocytes were cultured in serum-free medium in the presence of IFN-γ (2.5 ng/ml) or GM-CSF (100 ng/ml; M1 differentiation), with or without the PPAR-γ antagonist GW9662 (10 μM), for 4 days. Expression of CD206, CD163 and MerTK was measured by flow cytometry. **(B)** Pooled data are represented as mean values ± SEM. Analysis was performed using Student’s paired *t*-test. *P < 0.05; **P < 0.01; n.s., not significant. Data are representative of three independent experiments.

M1 stimulating cytokines such as IFN-γ and GM-CSF downregulate membrane expression of MerTK and CD163 [4]. IFN-γ also hinders dexamethasone induction of the M2c phenotype [33]. Unlike IFN-γ, GM-CSF is able to upregulate CD206 [4]. Herein, we investigated the effects of GW9662 in the presence of either IFN-γ or GM-CSF. We found that both IFN-γ and GM-CSF were permissive for GW9662 induction of the M2c receptor CD163, resulting in its significant upregulation. In parallel to what was reported above in the presence of IL-4 (Figure 1), in the presence of GM-CSF, GW9662 also led to significantly enhanced expression of CD206. However, in the presence of IFN-γ, CD206 expression did not increase, and neither IFN-γ nor GM-CSF allowed significant MerTK upregulation (Figure 2A-B). Therefore, similarly to what is observed for dexamethasone [33], the presence of M1 cytokines obstructs the effects of GW9662 on the M2c phenotype.

### GW9662 induces *M2c-like* cells that express MerTK and produce the MerTK ligand Gas6

**Figure 3:**
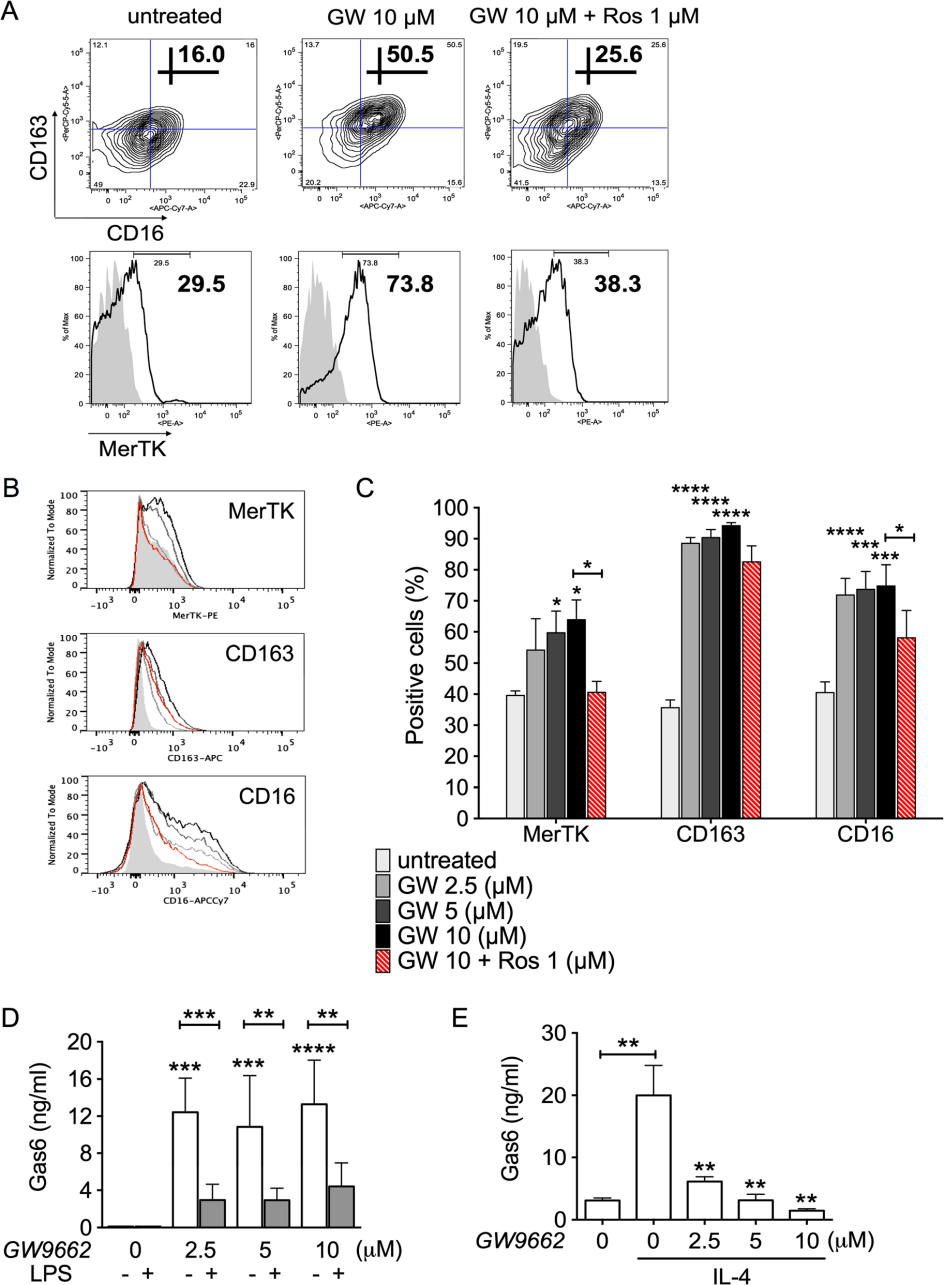
GW9662 induces *M2c-like* cells that upregulate MerTK and its ligand Gas6. **(A-C)** Healthy monocytes were cultured in serum-free medium in the absence of cytokines or growth factors (M0 differentiation), with or without the PPAR-γ antagonist GW9662 (2.5-10 μM), for 4 days; when specified, the PPAR-γ agonist rosiglitazone (1 μM) was added. Expression of MerTK, CD163 and CD16 was measured by flow cytometry. **(D-E)** Gas6 production levels were quantified by ELISA in culture medium, upon incubation with or without GW9662 (2.5-10 μM) of otherwise untreated cells (M0 conditions), LPS (50 ng/ml; M1 conditions) or IL-4 (20 ng/ml; M2a conditions) exposed cells. **(A-E)** Pooled data are represented as mean values ± SEM. Analysis was performed using one-way repeated measures ANOVA with Newman-Keuls multiple comparisons test. *P < 0.05; **P < 0.01; ***P < 0.001; ****P < 0.0001. When not specified by additional graphic signs, statistical annotations (asterisks) refer to comparisons with respect to the relative GW9662 untreated control group. Each set of data is representative of three independent experiments.

Subsequently, we examined the M2c polarizing effects of GW9662 on otherwise untreated cells (M0 conditions). Inhibition of PPAR-γ resulted in a dose-dependent upregulation of MerTK along with a strongly significant induction of the M2c-associated receptors CD163 and CD16 (Figure 3A-C). Addition of rosiglitazone (1 μM) exerted suppressive effects which were inversely proportional to the strength of GW9662 induction: in fact, rosiglitazone was able to neutralize the effects of GW9662 on MerTK and, partially, on CD16 expression, but failed to reverse the more robust upregulation of CD163 (Figure 3A-C).

Furthermore, we looked by ELISA at the effects of GW9662 on macrophage production of the MerTK ligand Gas6. Gas6 was previously shown to be released by both M2c and M2a macrophages [4]. In line with its role favoring M2c polarization, GW9662 strongly increased Gas6 protein levels in supernatants of otherwise untreated cells (Figure 3D). Of note, GW9662 induction of Gas6 was neutralized by LPS (Figure 3D), suggesting an inhibitory role of TLR signaling on Gas6 production. Remarkably, opposite effects were observed for GW9662 on Gas6 production in M2a conditions. In line with its inhibitory role on M2a polarization, in fact, GW9662 suppressed Gas6 production by IL-4 stimulated cells (Figure 3E).

Hence, GW9662 promotes differentiation of cells expressing the M2c (MerTK^+^ CD163^+^ CD16^+^) phenotype, so mimicking the effects previously observed with dexamethasone or with M-CSF plus IL-10 costimulation [4]. Accordingly, GW9662 stimulates Gas6 production by M2c differentiating cells, but inhibits Gas6 production by M2a differentiating cells.

### The PPAR-γ agonist rosiglitazone attenuates M2c polarization induced by dexamethasone

**Figure 4:**
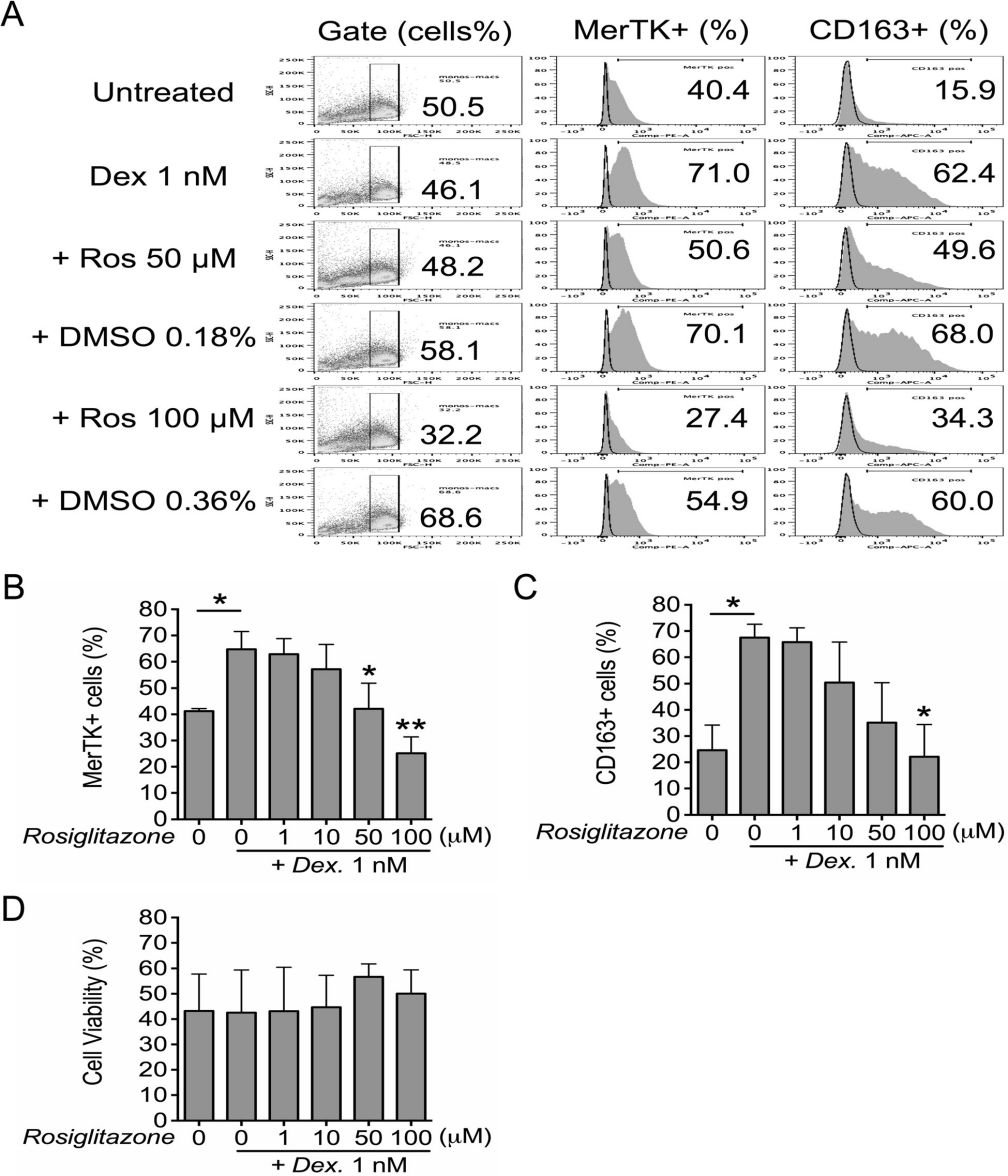
Rosiglitazone attenuates M2c polarization induced by dexamethasone. **(A-D)** Healthy monocytes were cultured in serum-free medium in the presence or absence of dexamethasone (1 nM; M2c differentiation), with or without the PPAR-γ agonist rosiglitazone (1–100 μM), for 4 days. Expression of MerTK and CD163 was measured by flow cytometry. Vehicle controls (DMSO > 0.15%) are shown in **(A)**. Percentages of cells with normal morphology according to forward (FSC) and side scatter (SSC) and contained in the gate of analysis depicted in **(A)** are shown in **(D)**. **(B-D)** Pooled data are represented as mean values ± SEM. Analysis was performed using one-way repeated measures ANOVA with Newman-Keuls multiple comparisons test and Student’s paired *t*-test. *P < 0.05; **P < 0.01. When not specified by additional graphic signs, statistical annotations (asterisks) refer to comparisons with respect to the relative rosiglitazone untreated control group. Data are representative of three independent experiments.

We cultured monocytes/macrophages in the presence of dexamethasone (M2c conditions), with or without the PPAR-γ agonist rosiglitazone. Consistent with the induction of M2c polarization by its antagonist GW9662, rosiglitazone (50–100 μM) was found to impede the upregulatory effects of dexamethasone (1 nM) on MerTK and CD163 expression (Figure 4A-C). Inhibitory effects were not due to vehicle toxicity (i.e., DMSO 0.18% for rosiglitazone 50 μM and DMSO 0.36% for rosiglitazone 100 μM) (Figure 4A), nor to significant effects of rosiglitazone on cell viability and morphology, as assessed by forward and side scatter by flow cytometry (Figure 4D). However, no significant inhibitory effects of rosiglitazone were seen in the presence of higher concentrations of dexamethasone (10 nM) (not shown) and/or lower doses of rosiglitazone (1–10 μM).

**Figure 5:**
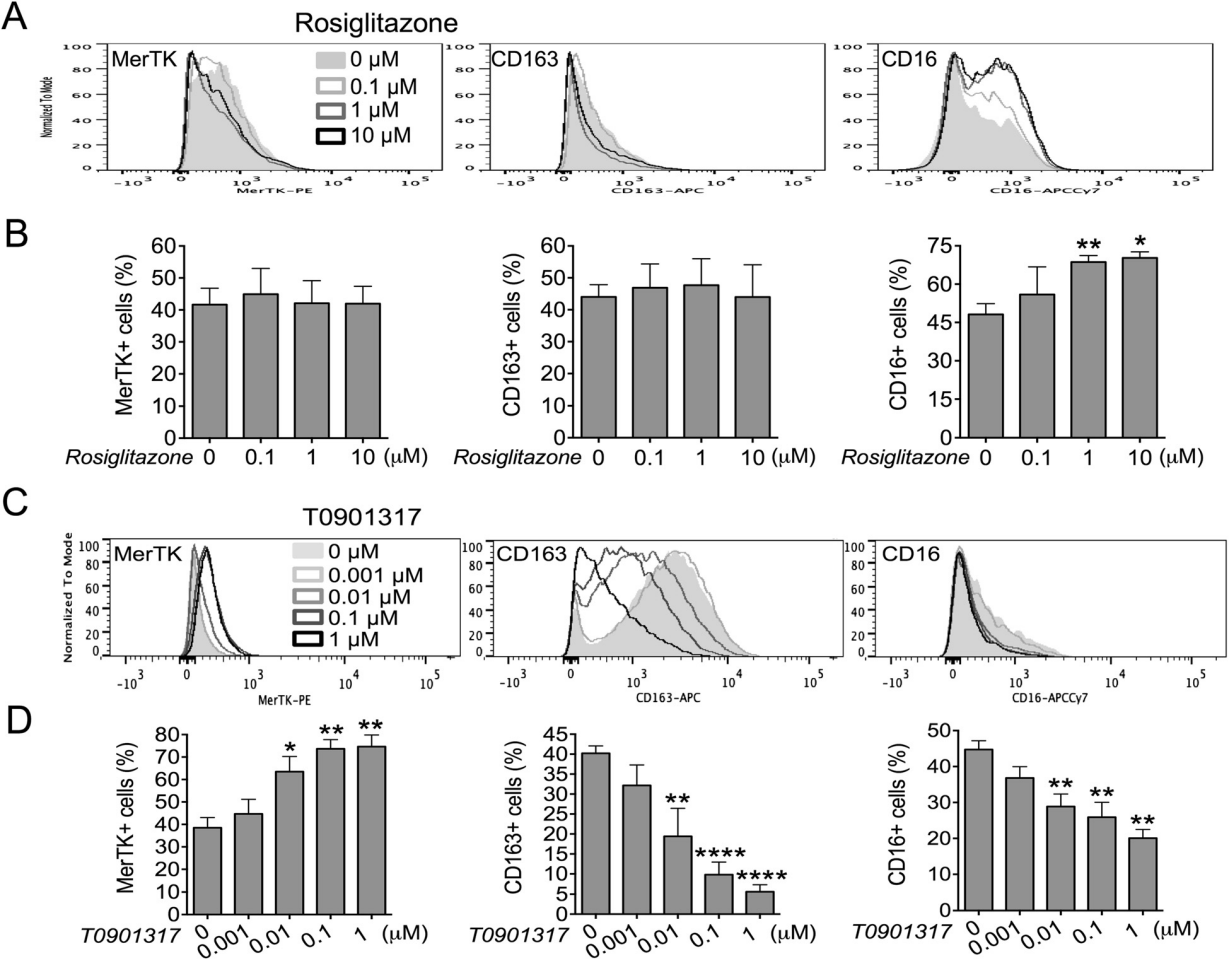
Rosiglitazone alone does not affect MerTK expression, whereas T0901317 upregulates MerTK regardless of M2c polarization. Healthy monocytes were cultured in serum-free medium with or without the PPAR-γ agonist rosiglitazone (0.1-10 μM) **(A-B)** or the LXR agonist T0901317 (0.001-1 μM) **(C-D)**, for 4 days. Expression of MerTK (*n* = 5 for either PPAR-γ or LXR stimulation assays), CD163 (*n* = 4) and CD16 (*n* = 3) was measured by flow cytometry. **(B and D)** Pooled data are represented as mean values ± SEM. Analysis was performed using one-way repeated measures ANOVA with Newman-Keuls multiple comparisons test and Student’s paired *t*-test. *P < 0.05; **P < 0.01; ***P < 0.001; ****P < 0.0001. Each set of data is representative of three to five independent experiments.

In otherwise untreated cells (M0 conditions), addition of rosiglitazone alone did not significantly change the expression of MerTK and CD163, although it modestly increased CD16 expression (Figure 5A-B).

### The LXR agonist T0901317 upregulates MerTK independently of M2c phenotype acquisition

To investigate the potential role of the nuclear receptors LXRs in M2c differentiation, we cultured monocytes in the presence or absence of the LXR agonist T0901317. In accord with results previously obtained in mice [20], T0901317 was found to upregulate MerTK expression, with significant effects already occurring at low doses (0.01 μM) (Figure 5C-D). Surprisingly, MerTK upregulation by T0901317 was not associated with the acquisition of the M2c phenotype. Indeed, the M2c surface markers CD163 and CD16 (Figure 5C-D), as well as the M2 receptor CD206 (not shown), were downregulated in a dose-dependent manner. Therefore, in contrast to what is observed for dexamethasone, M-CSF plus IL-10 [4] or GW9662, the MerTK expression pattern regulated by LXRs is uncoupled to M2c differentiation.

### Similarly to dexamethasone, GW9662 inhibits *in vitro* macrophage production of TNF-α and IL-10

**Figure 6:**
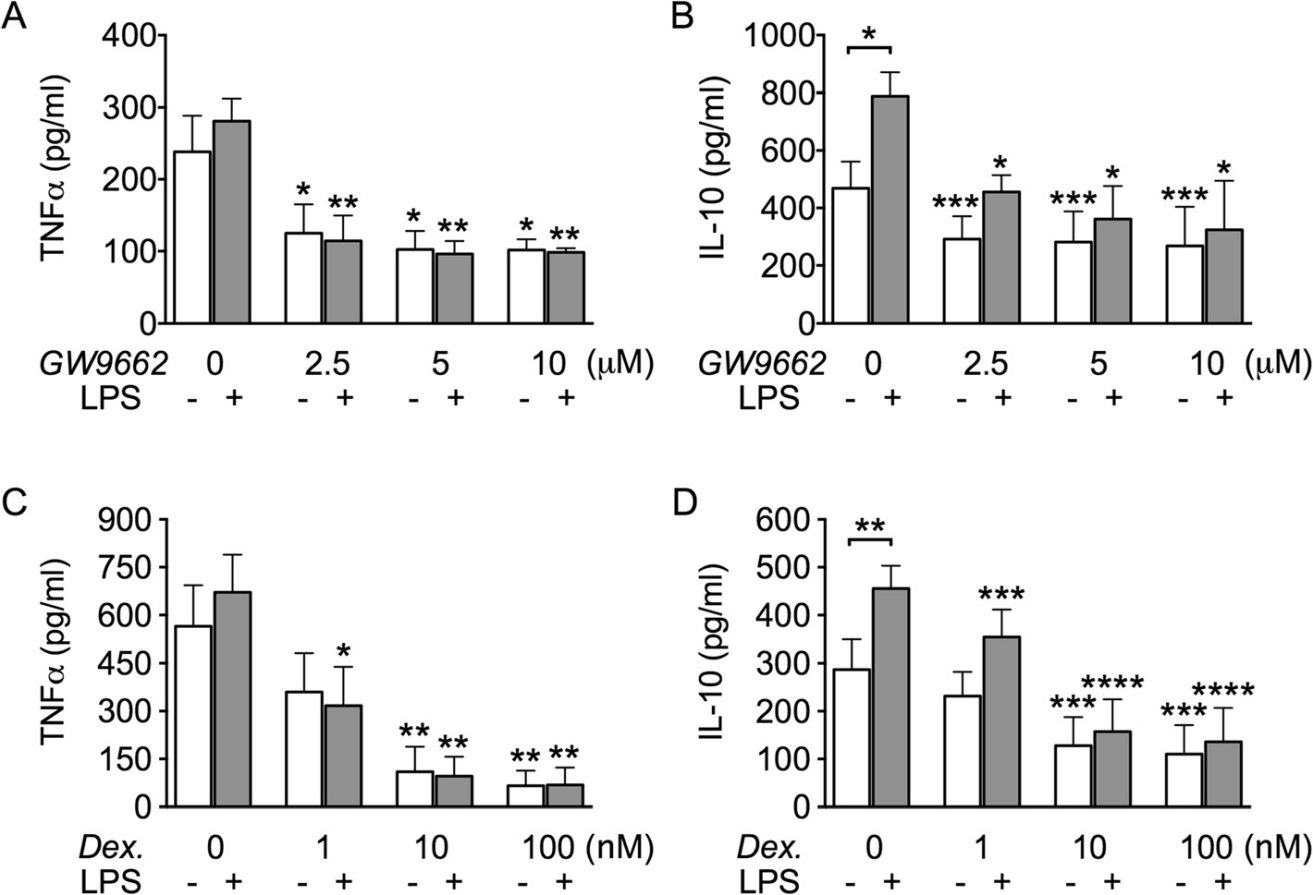
GW9662, like dexamethasone, inhibits *in vitro* production of TNF-α and IL-10. TNF-α **(A and C)** and IL-10 **(B and D)** production levels were quantified by ELISA in culture medium of cells incubated for 3 days in the presence or absence of GW9662 (2.5-10 μM) (*n* = 3) **(A and B)** or dexamethasone (1–100 nM) (*n* = 4) **(C and D)**. When specified, LPS (50 ng/ml) was added in the last 48 hours. Data are represented as mean values ± SEM. Analysis was performed using one-way repeated measures ANOVA with Newman-Keuls multiple comparisons test. *P < 0.05; **P < 0.01; ***P < 0.001; ****P < 0.0001. Comparison between spontaneous and LPS-triggered cytokine release was performed using Student’s paired *t*-test. When not specified by additional graphic signs, statistical annotations (asterisks) refer to comparisons respect to the relative GW9662 or dexamethasone untreated control. Each set of data is representative of three to four independent experiments.

Since GW9662 appears to stimulate the expansion of regulatory M2c cells, we sought to examine its effects on macrophage production of proinflammatory and anti-inflammatory cytokines. For this purpose, we measured by ELISA TNF-α and IL-10 levels released in supernatants of cells cultured with GW9662, without or after stimulation with low doses of LPS, and compared the effects with those obtained from dexamethasone cultures. In our conditions, LPS was able to significantly augment cell release of IL-10, but not TNF-α (Figure 6). GW9662 significantly reduced spontaneous as well as LPS-triggered release of both TNF-α (Figure 6A) and IL-10 (Figure 6B). Dexamethasone treatment yielded similar results (Figure 6C-D), in agreement with previous *in vitro* data [34,35].

Altogether, GW9662 and dexamethasone exert analogous effects: both induce differentiation toward the M2c phenotype, upregulate the MerTK/Gas6 pathway, and inhibit release of both TNF-α and IL-10 *in vitro*. With regard to cytokine production, GW9662 and dexamethasone driven cell populations differ from the M2c subset differentiated in the presence of M-CSF, which was instead previously shown to produce low levels of TNF-α but high levels of IL-10 *in vitro* [4].

### GW9662 does not enhance macrophage phagocytosis of apoptotic cells

**Figure 7:**
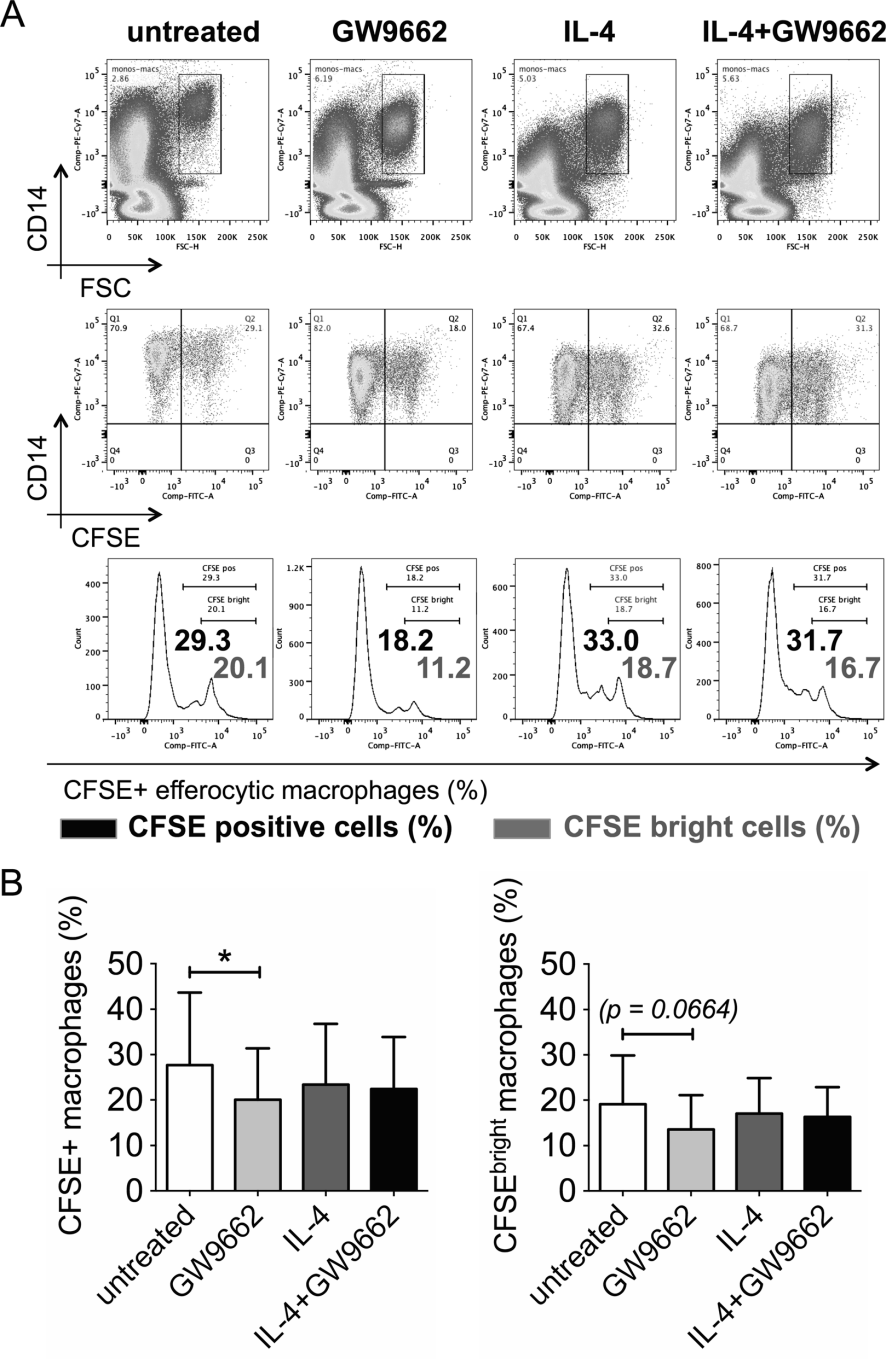
GW9662 does not enhance macrophage phagocytosis of apoptotic cells. **(A-B)** Apoptotic cells (ACs) were obtained by incubating healthy human neutrophils in 10% FBS-RPMI for 20 hours. CFSE-labeled ACs were added for 30 minutes, at a 5:1 ratio, to CD14-labeled macrophages cultured for 6 days in complete medium in the absence of cytokines (untreated; M0 differentiation) or in the presence of IL-4 (20 ng/ml; M2a differentiation), with or without GW9662 (10 μM). Percentages of total (CFSE^+^) and highly (CFSE^bright^) efferocytic macrophages are depicted. **(B)** Pooled data are represented as mean values ± SEM. Analysis was performed using Student’s paired *t*-test. *P <0.05. Data are representative of ten independent experiments.

Since conventional M2c macrophages, induced by dexamethasone or M-CSF and IL-10, are characterized by augmented capability to phagocytose ACs (efferocytosis) [4,33], we looked at the potential effects of GW9662 on macrophage phagocytosis of apoptotic neutrophils. For this purpose, CD14-labeled macrophages, differentiated in the presence or absence of IL-4 (20 ng/ml), with or without GW9662 (10 μM), were coincubated with CFSE-labeled apoptotic neutrophils at a 1:5 ratio for 30 minutes, and analyzed by flow cytometry. Consistent with previous studies supporting a central role for PPAR-γ in efferocytosis [26-29], we did not observe increased amounts of total (CFSE+) or highly (CFSE^bright^) efferocytic macrophages among GW9662-treated cells. Indeed, in otherwise untreated cells (M0 conditions), GW9662 significantly decreased efferocytosis, while in the presence of IL-4 (M2a conditions), no significant change was observed (Figure 7A-B). Therefore, GW9662-driven *M2c-like* cells differ from conventional M2c macrophages [4,33] because they do not show enhanced efferocytic properties.

## Discussion

In this paper, we investigated the role of PPAR-γ and LXR receptors in human M2 macrophage polarization, with particular focus on differentiation of the “M2c” anti-inflammatory subset. It has been reported that PPAR-γ mediates IL-4/STAT-6 effects on M2a (CD206^+^ CD209^+^) alternative macrophage activation [7,8,12,17,19], as well as GM-CSF effects on differentiation of M1-type and alveolar (CD206^+^ CD11c^hi^) macrophages [15,16,27], and the effects of both GM-CSF and IL-4 on differentiation of immature (CD209^+^ CD1a^−^) dendritic cells [17,36]. Here we show that inhibition of PPAR-γ during monocyte-to-macrophage maturation, by means of the PPAR-γ antagonist GW9662, elicits differentiation of cells carrying the M2c phenotype (CD206^high^ CD209^−^ CD163^+^ CD16^+^ TNF-α^−^) and upregulates the MerTK/Gas6 pathway. In the presence of IL-4 (M2a conditions), GW9662 amplifies CD206 expression, downregulates CD209, and upregulates CD163 and MerTK, thereby producing a phenotypic M2a-to-M2c shift. Similar to what we observed for dexamethasone-induced M2c differentiation [33], IFN-γ, GM-CSF or LPS (M1 conditions) impede GW9662 upregulation of MerTK and Gas6, although GW9662 still reverses CD163 downregulation provoked by M1 cytokines and - as occurs with IL-4 - it further amplifies CD206 expression induced by GM-CSF. In opposition to the M2c polarizing effects of GW9662, the PPAR-γ agonist rosiglitazone attenuates MerTK and CD163 upregulation occurring in the presence of dexamethasone (M2c conditions). Like dexamethasone, GW9662 also inhibits macrophage *in vitro* production of TNF-α and IL-10. But, unlike dexamethasone-induced M2c cells, GW9662-induced *M2c-like* cells do not show enhanced phagocytosis of ACs; rather, efferocytosis is impaired, in accordance with the central role previously reported for PPAR-γ in the clearance of ACs [26-29]. In the case of IL-4-treated cells, addition of GW9662 does not exert net effects on AC phagocytosis, suggesting that, in M2a-to-M2c shifted macrophages, inhibition of PPAR-γ-dependent efferocytic pathways, mediated by CD36, thrombospondin-1, transglutaminase-2, AXL, pentraxin-3 and/or immunoglobulin receptor FcγRI [26-29], might be compensated by enhanced MerTK-dependent efferocytosis [4,5].

LXRs are known to mediate MerTK expression in murine macrophages exposed to ACs. Cholesterol and oxysterol species contained in ingested ACs drive LXR induction of MerTK, resulting in enhancement of AC clearance and transrepression of macrophage inflammation in response to phagocytosis [20]. In human macrophages, we confirm that the LXR agonist T0901317 upregulates MerTK. However, LXR induction of MerTK is unexpectedly dissociated from acquisition of the M2c phenotype; indeed, CD206, CD163 and CD16 are downregulated. In light of these data, we hypothesize that MerTK regulation follows at least two expression patterns: one linked to the M2c phenotype, driven by M-CSF and IL-10, glucocorticoids or PPAR-γ antagonists; and another one independent from M2c polarization, driven by LXRs through AC-derived oxysterols and non-steroidal LXR agonists.

The present study adds to our previous research on characterization of anti-inflammatory M2c macrophages in humans [4,33]. We recently described the M2c subset as CD206^+^ CD163^+^ CD16^+^ MerTK^+^ M2 macrophages, well distinguished from IL-4-induced CD206^+^ CD209^+^ M2a cells, able to release high levels of the MerTK ligand Gas6 and specialized in phagocytosis of early apoptotic cells *via* MerTK [4]. Differentiation of monocyte-derived macrophages in the presence of M-CSF plus serum, M-CSF plus IL-10, or glucocorticoids gives rise to the M2c phenotype [4]. However, some differences exist among different stimulations. For instance, M2c cells induced by M-CSF and IL-10 highly express CD14, while dexamethasone-driven M2c cells express CD14 levels comparable to baseline. Moreover, M2c cells obtained in the presence of M-CSF produce high levels of IL-10, which are further amplified by Gas6 *via* MerTK [4], whereas dexamethasone, as shown here, inhibits IL-10 production *in vitro*. Although classified as M2c stimuli [2], we previously observed that TGF-β gives a different phenotype, characterized by CD206 and CD16 induction, but inhibition of CD163, MerTK and Gas6, whereas IL-10 without M-CSF gives only a partial phenotype, characterized by upregulation of CD163 and Gas6, but not CD206, CD16 and MerTK [4]. Herein, we further describe a new variant of *M2c-like* cells induced by GW9662, reporting similarities and differences with conventional M2c subsets. Such heterogeneity among macrophage populations recently spurred the proposal to adopt in the near future revised macrophage nomenclature more strictly linked to the activation standards [37].

Other authors previously investigated GW9662 and rosiglitazone effects on human macrophage phagocytosis of ACs and phenotype. In agreement with our findings, Bouhlel and colleagues [19] observed that rosiglitazone alone did not affect macrophage differentiation in basal conditions, yet it amplified M2a polarization induced by IL-4, including CD163 downregulation. In accord with our results, Majai and colleagues [28] found that GW9662 decreased AC phagocytosis, owing to downregulation of efferocytic molecules such as CD36, transglutaminase-2 and AXL, and inhibited LPS-induced IL-10 production; also, dexamethasone was shown to share with GW9662 down-regulation of CD36, transglutaminase-2 and IL-10. In contrast with our observations, however, these authors failed to find significant changes in MerTK, Gas6, CD206 or CD16 expression upon GW9662 treatment, nor did they observe GW9662 inhibition of TNF-α production. It is important to note that Majai *et al.* [28] cultured monocytes in medium containing M-CSF and human serum, a combination that we demonstrated already promotes M2c polarization *in vitro* [4]; thus, their experimental conditions may have masked the M2c polarizing effects of GW9662 that we observed. Our finding that PPAR-γ inhibition leads to differentiation of *M2c-like* monocytes/macrophages is then novel, and may have significant implications in several fields focusing on macrophage biology, including studies on adipose tissue-associated macrophages, macrophages of atherosclerotic plaques, macrophage activation in chronic inflammatory diseases like systemic lupus erythematosus (SLE), and tumor-associated macrophages.

### PPAR-γ and M2 polarization in adipose tissue-associated macrophages (ATMs)

PPAR-γ agonists of the thiazolidinediones (TZDs) class, like rosiglitazone, are currently used to treat type-2 diabetes mellitus patients, mainly due to their insulin-sensitizing effects. Macrophage-specific deletion of PPAR-γ results in reduced numbers of adipose tissue-associated macrophages (ATMs) in white adipose tissue, impaired M2a polarization and defective oxidative metabolism. Macrophage dysregulation would, in turn, predispose to diet-induced obesity, reduced production of adiponectin, glucose intolerance and insulin resistance [8]. Previous studies demonstrated that in both mice and humans, ATMs from obese subjects express a mixed phenotype, in mice referred from some authors as “M2b” [38], characterized by M2 markers (e.g., CD206, IL-10) and M1 proinflammatory cytokines (e.g., TNF-α) [38,39]; PPAR-γ is upregulated in these macrophages, and rosiglitazone can inhibit TNF-α production, thereby promoting a phenotypic M2b-to-M2a shift [38]. Other authors showed that ATMs from obese mice include M2a-type Macrophage Galactose binding Lectin-1^+^ (MGL-1^+^) macrophages located in interstitial spaces, and M1-type CD11c^+^ macrophages surrounding necrotic adipocytes [40]. High-fat diet (HFD) induces higher expression in MGL-1^+^ cells of M2a markers (i.e., STAT-6) and lower expression of M2b (i.e., SPHK-1) and M2c markers (i.e., CD163, IL-10), along with increased levels of M1 markers (i.e., IL-12p40) [41,2]. Prolonged HFD finally elicits the expansion of cells with a mixed M2a/M1 double-positive MGL-1^med^/CD11c^+^ phenotype (IL-13^+^ STAT-6^+^ IL-12p40^+^), which express lower levels of inducible nitric oxide synthase (iNOS) and IL-1β compared to pure M1 cells, upregulate PPAR-γ coactivators and are further expanded following administration of TZDs [41]. Altogether, PPAR-γ appears to drive positive effects on oxidative metabolism, adipogenesis and tissue remodeling in response to obesity, in part related to M2a polarization, but it still allows chronic low-grade macrophage inflammation, due to persistence of CD11c^+^ IL-12p40^+^*M1-like* profile and paucity of regulatory CD163^+^ IL-10^+^ M2c cells. The M2c expansion that we observed by antagonizing PPAR-γ is consistent with this notion. In this regard, it is noteworthy that although PPAR-γ agonists were shown to inhibit macrophage production of proinflammatory cytokines like TNF-α and IL-6 induced by PMA [23,24], probably owing to inhibition of PKC-α membrane translocation [42], TZDs failed to suppress LPS-induced production of TNF-α and IL-6 [24], indeed production was even increased [43].

### PPAR-γ and M2 polarization in macrophages of atherosclerotic plaques

The role of PPAR-γ in macrophages from atherosclerotic lesions is controversial. TZDs were shown to inhibit atherogenesis in LDL-R KO and ApoE KO mouse models [44,45] and to reduce carotid artery wall thickness in diabetic patients [46]. However, *in vivo* impact of TZDs in atherosclerosis depends on mechanisms involving multiple cell targets apart from macrophages, such as inhibition of endothelial activation [45], inhibition of vascular smooth muscle cell proliferation [47], reduction of vascular resistance and blood pressure [48], increased insulin sensitivity and adiponectin production [8], and anti-oxidant properties [49]. Moreover, although PPAR-γ may limit atherogenesis at initial phases, serious doubts arise about its role in plaque instability. In fact, rosiglitazone was ultimately reported to increase the risk of myocardial infarction in diabetic patients [50,51], leading to its withdrawal from the market in several countries. Plaque instability is favored by enlargement of necrotic core in atherosclerotic lesions. Cholesterol-laden macrophages undergo apoptosis, and apoptotic macrophages turn into secondary necrotic cells if not promptly cleared [52,53]. Macrophage-specific PPAR-γ might have atherogenic potential by driving phagocytosis of oxLDLs *via* CD36. PPAR-γ is in fact inducible by oxLXLs themselves, and is expressed in *M1-like* macrophage foam cells of human atherosclerotic lesions [23]. On the other side, LXRs, MerTK and CD163 seem to prevent plaque instability. LXRs protect against foam cell formation, by inducing ABC transporter-mediated cholesterol efflux [9] and by upregulating MerTK in mice [20] and in humans (as shown in this paper). MerTK, in turn, inhibits uptake of lipoproteins [54] shifting phagocytosis activity toward efficient and non-inflammatory clearance of cholesterol-laden apoptotic macrophages [52]. Additionally, both LXRs and MerTK exert anti-apoptotic effects on macrophages [31,33,55]. CD163 exerts beneficial effects owing to upregulation of heme oxygenase-1 in response to hemoglobin-haptoglobin complexes, which ultimately results in iron clearance and prevention of oxidative reactions, along with release of IL-10 and anti-inflammatory heme metabolites [56]. LXR-α and MerTK were demonstrated to be atheroprotective in LDL-R KO and ApoE KO mouse models [52,53,57]. In humans, non-foamy protective *M2-like* CD206^+^ macrophages expressing high levels of MerTK [26,58] and CD163 [59,60] have been described in areas of plaques far from the necrotic core and close to microvessels or microhemorrhages, respectively. We hypothesize that the potential atherogenic role of PPAR-γ may become explicit in the presence of IL-4 or other PPAR-γ agonists like TZDs. IL-4 can in fact amplify PPAR-γ expression induced by oxLDLs [13], and at the same time down-regulate LXR expression, so that PPAR-γ activation of LXRs is impaired [26]. In this setting, macrophage uptake of lipoproteins is not followed by cholesterol efflux, thereby facilitating foam cell formation. Moreover, chronic stimulation with IL-4 and PPAR-γ activation induce apoptosis in macrophages [33,61], while IL-4 down-regulation of MerTK and CD163 [4] may interfere with the clearance of apoptotic macrophages and iron, respectively. In fact, in both LDL-R KO and ApoE KO mice, IL-4 proved to extend the size of atherosclerotic lesions [62,63]. Taking together our present findings and previously reported data, we suggest that new PPAR-γ agonists not affecting macrophage-specific PPAR-γ might overcome controversial effects and cardiovascular safety concerns of TZDs. On the other hand, treatments apt to elicit the expansion of MerTK^+^ and CD163^+^ cells (e.g., M2c polarizing agents and IL-4/STAT-6 inhibitors) may help against atherosclerosis progression.

### PPAR-γ and M2 polarization in systemic lupus erythematosus (SLE)

Pathogenic macrophage populations in SLE are traditionally considered to be M1 oriented [64], in accord with increased production of IFN-γ during disease exacerbations [65]. More recently, SLE macrophages were classified as M2b cells [66]. In addition to enhanced expression of iNOS and proinflammatory cytokines, in fact, they also express high levels of IL-10 and relatively low levels of IL-12 [66]. Excessive immune complexes and TLR signaling occurring in SLE may in fact stimulate M2b polarization [2], which may partially account for increased IL-10 levels in SLE serum [65]. By contrast, serum levels of IL-4 are not significantly changed in SLE, and M2a macrophages do not seem to be expanded [64,65]. We and others recently reported higher circulating levels of MerTK and CD163 cleavage products in active SLE patients, thereby suggesting increased turnover of M2c cells and relative insufficiency of M2c and MerTK activity [32,67,68]. In fact, lupus-like syndromes arise because of defective clearance of ACs owing to impaired expression or function of efferocytic molecules, as occurs in mice lacking functional MerTK [69]. The therapeutic utility of glucocorticoids in SLE may be at least in part attributable to M2c induction, MerTK upregulation and enhanced macrophage phagocytosis of ACs [4,5,67]. Previous studies support a protective role for both LXRs and PPAR-γ in SLE: LXR-α/β and PPAR-γ KO mice accumulate ACs in spleens, produce anti-nuclear autoantibodies and develop lupus-like syndrome with glomerulonephritis [20,29]. Also, TZDs were reported to ameliorate murine lupus [48,70,71]. However, beneficial effects of TZDs were only observed at early stages and were mostly associated with vascular and insulin-sensitizing effects (i.e., inhibition of endothelin production, vasodilation, reduced blood pressure, improvement of lipid metabolism) rather than to direct anti-inflammatory effects on macrophages [48,70,71]. Likewise, reduced renal inflammation and reduced macrophage activation appeared to be an indirect effect secondary to adiponectin induction [71]. Although a rationale for using TZDs in SLE autoimmunity would be conversion of M2b macrophages into M2a cells [38] and promotion of PPAR-γ-dependent efferocytosis [26-29], the inhibitory role herein shown on M2c differentiation and on MerTK expression should be taken into account.

### PPAR-γ and M2 polarization in tumor-associated macrophages (TAMs)

Tumor-associated macrophages (TAMs) are generally considered *M2c-like* macrophages, expressing CD206 and CD163 [72]. As M2c cells, TAMs also release Gas6, which facilitates tumor cell proliferation and probably immune tolerance to cancer [4,73]. Besides direct anti-proliferative effects on certain tumor cells [23], PPAR-γ may exert anti-tumoral effects by acting on macrophages through a dual mechanism. On the one hand, PPAR-γ inhibits inflammation-driven carcinogenesis by suppressing NF-κB and proinflammatory genes in *M1-like* cells. On the other hand, PPAR-γ restores anti-tumor cytotoxic T lymphocyte activity by inhibiting tolerogenic *M2c-like* TAMs [74]. Our finding that GW9662 inhibition of PPAR-γ generates *M2c-like* macrophages and Gas6 release is in fact consistent with previously reported GW9662 suppression of anti-tumor immune responses [74].

## Conclusions

This study extends our knowledge of the role of PPAR-γ and LXRs receptors in human macrophage activation. We show that blocking PPAR-γ during monocyte-to-macrophage maturation elicits differentiation of *M2c-like* CD206^+^ CD163^+^ cells and upregulation of the MerTK/Gas6 axis. Although PPAR-γ signaling may reduce M1 (and M2b) inflammatory cytokine production and potentiate M2a alternative activation, results suggest that it also impedes M2c polarization and restoration of fully anti-inflammatory conditions in chronic settings like metabolic syndrome and autoimmune diseases. Therapeutic advantages might derive from the use of more selective PPAR-γ agonists, targeting adipose tissue PPAR-γ2 isoform rather than ubiquitously expressed PPAR-γ1 isoform [75], with the aim of exploiting beneficial effects of PPAR-γ on insulin-sensitivity and adiponectin secretion while minimizing controversial effects on macrophages. Finally, we point out that LXR stimulation upregulates MerTK independently of M2c polarization, thus revealing the existence of different regulation patterns for MerTK expression.
